# Cuttlefish Bone-Derived Calcium Phosphate Bioceramics Have Enhanced Osteogenic Properties

**DOI:** 10.3390/jfb15080212

**Published:** 2024-07-29

**Authors:** Boqi Pang, Jiaru Xian, Jiajun Chen, Liqi Ng, Mengting Li, Guangchun Zhao, Yixun E, Xiaorui Wang, Xiaxin Cao, Changze Zhang, Mingjing Zhang, Chaozong Liu

**Affiliations:** 1 Hainan Provincial Fine Chemical Engineering Research Center, Hainan University, Haikou 570228, China; 2 Institute of Orthopaedic & Musculoskeletal Science, University College London, Royal National Orthopaedic Hospital, London HA7 4LP, UK

**Keywords:** cuttlefish bones, calcium phosphate bioceramics, osteogenesis

## Abstract

Cuttlefish bones are byproducts of cuttlefish processing and are readily available in the marine food industry. In this study, calcium phosphate bioceramics were prepared from cuttlefish bones using a two-stage hydrothermal calcination process. The results indicated that all bioceramics derived from cuttlefish bones had a higher degradation capacity, better bone-like apatite formation ability, and higher degree of osteogenic differentiation than commercially available hydroxyapatite. Notably, β-tricalcium phosphate, which had the highest degree of Ca^2+^ and Sr^2+^ dissolution among the bioceramics extracted, can significantly upregulate osteogenic markers (alkaline phosphatase, osteocalcin) and stimulate bone matrix mineralization. Thus, it is a promising bioceramic material for applications in bone regeneration.

## 1. Introduction

Critical-size bone defects caused by severe trauma, infection, tumor removal, and congenital diseases are managed surgically using bone transplantation [1,2]. Autologous bone grafts are considered the gold standard for bone-defect repair owing to their nonimmunogenicity and efficient bone regeneration [3]. However, their use is restricted by procurement morbidity, a lack of availability, and their rapid resorption [4,5]. Calcium phosphate bioceramics (CaPs) are bone tissue substitutes that play a prominent role in biomaterial implantation [6]. Depending on the physical properties required for a particular application, different phases and forms of CaPs, including hydroxyap-atite (HA), α-tricalcium phosphate (α-TCP), β-tricalcium phosphate (β-TCP), biphasic calcium phosphate (BCP), and monocalcium phosphate monohydrate (MCP), are currently used in the biomedical industry [7]. Chemical synthesis of CaPs requires the use of harsh chemicals, is expensive and time-consuming, and generates undesirable byproducts [8,9]. Compared with synthetic compounds, CaPs synthesized from natural sources have superior biocompatibility and osteoconductivity as they contain the trace elements necessary for rapid bone regeneration [10]. Additionally, extraction from natural resources is an eco-friendly and sustainable process [11]. However, certain natural sources, such as mammalian sources, pose a high risk of infection and disease transmission. A specific commercial xeograft derived from bovine bone called Bio-Oss^®^ (Geistlich Pharma AG, Wolhusen, Switzerland) (ABB) has been widely used in clinical applications and investigated by various research institutions [12,13]. However, some believe that the products of Bio-Oss^®^ made of bovine bones carry the risk of infectious disease transmission [14].

Cuttlefish bone contains several trace elements, including sodium, magnesium, potassium, and strontium, which have a high capacity to accelerate bone formation. Furthermore, it is an inexpensive, widely available raw material consisting of 84% CaCO_3_, which can be readily transformed into HA and BCP [15]. Cozza et al. [16] found that HA synthesized from cuttlefish bone stimulated cell proliferation and differentiation more effectively than stoichiometric HA. Rocha et al. [17] transformed cuttlefish bones hydrothermally into HA tissue scaffolds retaining the cuttlebone architecture and found that the channel sizes of the cuttlebone samples investigated were beneficial for bone growth (~100 × 200 μm). Ivankovic et al. [18] prepared highly porous HA through the hydrothermal transformation of aragonitic cuttlefish bones at temperatures ranging from 140 to 220 °C for different periods ranging from 20 min to 48 h. They reported that three-dimensional (3D) structures based on the natural morphology of cuttlefish bones were promising alternatives for bone tissue engineering applications. Cuttlefish bones can also be used to generate BCP bioceramics. Sarin et al. [19] converted cuttlefish bones into porous BCP scaffolds and found that tailoring the composition of these BCP scaffolds allowed the development of implants with controlled biodegradation, and their superior mechanical and microstructural properties can benefit efficient osteointegration and osteoinduction. Neto et al. [20] obtained cuttlefish bone-derived BCP scaffolds with different ions (Sr^2+^, Mg^2+^, and Zn^2+^) incorporated via hydrothermal transformation and calcination. These doped ions form inorganic minerals with good biocompatibility in the scaffold and promote cell proliferation, differentiation, and ossification. Therefore, cuttlefish bones may be an ideal bone repair material.

The objective of this study was to investigate the conversion of natural cuttlefish bone into various CaPs, along with their osteogenic properties. The chemical compositions, morphologies, mineralization capacities, and degradation rates of calcium phosphate bioceramics extracted from natural cuttlefish bones (C-CaPs) were evaluated. The biodegradability of the CaPs was characterized by weight loss in phosphate-buffered saline (PBS). The bioactivity was evaluated by observing and analyzing the formation of apatite on the surface of simulated body fluid (SBF). Furthermore, the biological performance was assessed by evaluating L929 cell adhesion and prolifer-ation, as well as the osteogenic differentiation of bone marrow-derived mesenchymal stem cells.

## 2. Materials and Methods

### 2.1. Materials

Phosphate acid (Xilong Scientific, Shantou, Guangdong, China, AR), 30% hydrogen peroxide (Xilong Scientific, Shantou, Guangdong, China, AR), and commercial HA (RHΛWN, biomedical grade, ≥98%) were used in this study.

### 2.2. Synthesis of C-CaPs from Cuttlefish Bone

**Figure 1:**
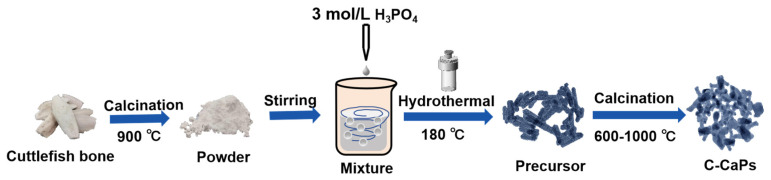
Synthesis of C-CaPs.

Native cuttlefish bones from Hainan were ground into powder and heated at 900 °C for 5 h to convert the calcium carbonate into calcium oxide. The prepared powder was dispersed in deionized water and subjected to ultrasonic treatment for 10 min. -Phosphoric acid (H_3_PO_4_) was added to the suspension, followed by stirring for 3 h and hydrothermal treatment 180 °C for 10 h. The mixture was then separated into solid and liquid phases, yielding the precursor. Subsequently, calcination was performed at different temperatures (600, 700, 800, 900, and 1000 °C) at a heating rate of 10 °C/min in air. Once the calcination temperature was reached, the bones were maintained isothermally for 2 h and then cooled at 20 °C/min. The material obtained via calcination at 600 °C was HA (C-HA); that obtained at 700, 800, and 900 °C was BCP, and that obtained above 1000 °C was β-tricalcium phosphate (C-βTCP). The powder obtained via -calcination at 700 °C was selected as the C-BCP group for further study. A schematic of the C-CaP synthesis process is shown in Figure 1.

### 2.3. Characterization

The phase was evaluated via X-ray diffraction (XRD) analysis (Smart Lab, Rigaku, Japan) using monochromated Cu-Kα radiation over an angular range of 20°–60°, with a step size of 0.01, a scan speed of 10°, a 40 kV voltage, and a 30 mA current. The relative amount of HA was calculated using the relative areas of the three strong characteristic peaks. The following formula was used [11,21]:

(1) $$w_{1}=\frac{I_{1}}{I}$$

where *I*_1_ represents the reflection intensity of three characteristic peaks of HA or β-TCP, *I* represents the sum of the reflection intensities of the three characteristic peaks of both HA and β-TCP, and *w*_1_ represents the relative amount of HA or β-TCP in the sample.

The functional groups were identified using Fourier transform infrared (FTIR) spectroscopy (TENSOR27, Bruker, Karlsruhe, Baden-Württemberg, Germany). Sample spectra were averaged over three scans, ranging from 500 to 4000 cm^−1^ at a resolution of 4 cm^−1^. The surface morphology was examined using optical and scanning electron microscopy (SEM; GeminiSEM 300, Zeiss, Oberkochen, Baden-Württemberg, Germany) coupled with energy-dispersive X-ray spectroscopy (EDS) for elemental analysis. The crystallography of the samples was investigated using transmission electron microscopy (JEM-2100F, JEOL, Akishima-shi, Tokyo Metropolis, Japan). The samples were dispersed in ethyl alcohol via ultrasonication for 15 min before being tested on a copper grid.

### 2.4. In Vitro Degradation Test

The biodegradability of the samples was examined in a shake PBS solution (pH = 7.4) at 37 °C. The control group was commercial HA, and the experimental group was C-HA, C-BCP, and C-βTCP. Three samples for each group were weighed as w_0_ and immersed in the shake PBS solution at 37 °C for 7, 14, 21, and 28 d. The weight loss (%) was determined using the following formula: weight loss (%) = (w_0_ − w_1_)/w_0_ × 100%, where w_0_ and wt represent the dry weights of the powder before and after immersion, respectively. The pH values of the biodegradation medium in all the remaining test tubes were measured using a pH meter (Mettler Toledo) during biodegradation. In addition, the release of Ca^2+^, ${\mathrm{PO}}_{4}^{3-}$, and Sr^2+^ ions from the samples was measured using inductively coupled plasma atomic emission spectroscopy (ICP-AES; Varian Inc., Palo Alto, CA, USA).

### 2.5. In Vitro Mineralization Test

SBF was prepared using the method described by Kokubo [22]. The bioactivity of the materials was assessed by immersing them in a shake 2× SBF solution at 37 °C for 14 d and testing the growth of apatite on their surface. The SBF was replaced every 7 d. The surface development of apatite was investigated using SEM. Additionally, FTIR spectroscopy was used to examine the variations in the functional groups prior to and following mineralization, and XRD was used to evaluate the crystal phase alterations.

### 2.6. Cell Viability Assay

The toxicity of the cuttlefish bone-derived bioceramics was assessed using L929 cells obtained from the Shanghai Cell Bank of the Chinese Academy of Sciences. The cells were cultured in Dulbecco’s modified Eagle’s medium (DMEM) supplemented with 10% fetal bovine serum (FBS) and 1% penicillin-streptomycin solution. The culture media was placed in an incubator at a temperature of 37 °C, with a humidity level saturated with 5% CO_2_. The cells were cultivated until they reached 80% confluence in order to be used for further tests. 

The cytotoxicity of the cuttlefish bone-derived bioceramics was evaluated using the CCK-8 (Dojindo, Kumamoto, Japan) assay with L929 cells. Briefly, cells were seeded into 24-well plates at a density of 1 × 10^4^ per well and incubated overnight. The samples (C-HA, C-BCP, C-βTCP) with different concentrations (200, 400, 600, and 800 µg/mL) were added to a 24-well plate. After incubation at 37 °C for 24 h, the culture medium was removed, and CCK-8 was added to each well. After 2 h, the formazan crystals were dissolved in dimethyl sulfoxide (DMSO), and the absorbance at 450 nm was measured using a microplate reader (Singapore, model: Mutiskan Sky). The proportion of the control group without sample treatment was used to express the cell viability. In order to assess cell proliferation, live/dead staining was used. The L929 cells were treated with various substances at a concentration of 800 µg/mL for 24 h. Following this, the cells were washed three times with PBS and stained for 10 min using solutions of propidium iodide (PI; Sigma–Aldrich) and acridine orange (AO; Sigma–Aldrich, St. Louis, MO, USA). At last, the samples were stained and examined using a Leica DM50000B (Leica Microsystems, Wetzlar, Germany) fluorescence microscope.

### 2.7. Osteogenic Potential Analyses

#### 2.7.1. Preparation of Conditioned Medium

The osteogenic ability of different samples (C-HA, C-BCP, C-βTCP, and commercial HA) was measured via alkaline phosphatase (ALP) staining and Alizarin Red staining assays. Before extraction, the samples were sterilized in 70% ethanol and exposed to ultraviolet light for 30 min. The sterilized samples were immersed in DMEM (Thermo Fisher Scientific, Waltham, MA, USA) at 37 °C for 24 h. Subsequently, the samples were centrifuged at 5000 rpm for 10 min, and the supernatants were collected and filtered through a 0.22-μm filter to obtain the conditioned medium (CM, 200 mg/mL).

#### 2.7.2. Osteogenic Differentiation and Mineralization In Vitro

Rabbit bone marrow-derived mesenchymal stem cells (rBMSCs) were seeded in a 6-well plate and cultured in a regular culture medium. After 12 h, the regular medium was replaced with an osteoinductive medium containing 50% conditioned medium. After 7 d of culture, ALP secreted from cells in the presence of bioceramic extracts was evaluated using an ALP staining kit in accordance with the manufacturer’s instructions, and the stained cells were photographed under a biological light microscope (model HP31). The positively stained area was calculated using ImageJ software (V1.8.0.112). Furthermore, an Alizarin Red S assay was performed to investigate calcium deposition. After 14 d, the medium was removed from the cells, and the cells were rinsed with PBS. Subsequently, the cells were immobilized with 4% paraformaldehyde (Merck, Darmstadt, Germany) for 20 min and stained with a 40 mM Alizarin Red (Sigma, USA) solution (pH 4.2) for 10 min. The cells were then rinsed with PBS and imaged under a microscope.

#### 2.7.3. Quantitative Real-Time PCR Analysis

The expression of osteogenic differentiation genes (ALP and osteocalcin (OCN)) was determined using a real-time quantitative polymerase chain reaction (RT-qPCR) assay. After 7 d of osteogenic culture, the total RNA was extracted, and the OD value of RNA was measured using an ultrafine nucleic acid protein tester (scandrop100) at an A260/A280 ratio. Reverse transcription was performed using the FOREGENE Corporation reverse transcription kit (RTOR-EasyTM II). The expression of osteogenic markers was quantified using the SYBR^®^Green Realtime PCR Master Mix (Osaka, Japan). GAPDH served as an internal reference. The comparative threshold cycle method was used to analyze the qPCR results using CFX Manager Software 3.1 (Bio-Rad, Hercules, CA, USA), with GAPDH as the reference gene. The primer sequences used for the qPCR are presented in Table 1.

## 3. Results and Discussion

### 3.1. Compositions and Morphologies of Cuttlefish Bone-Derived C-CaPs

**Figure 2:**
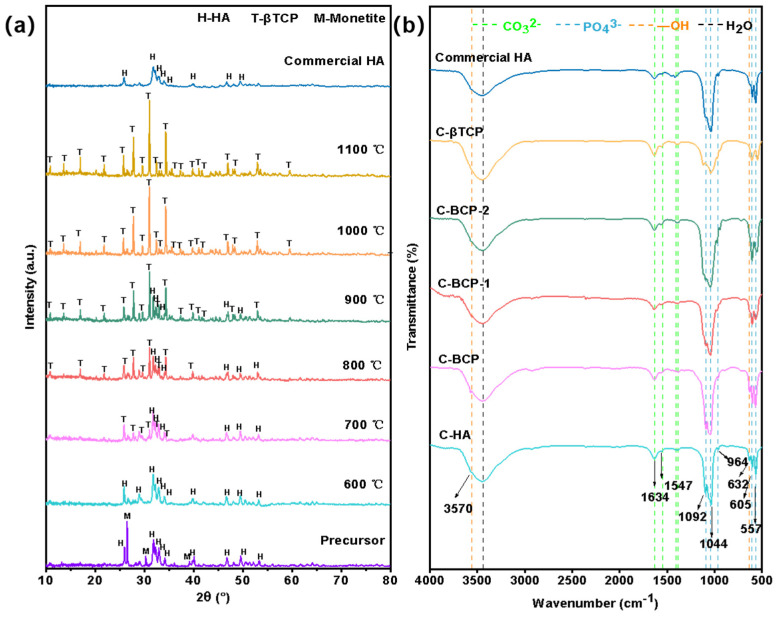
Physicochemical structure characterizations of the prepared samples. (**a**) XRD patterns of the precursor, the commercial HA, and the C-CaPs obtained at various temperatures. (**b**) FTIR spectra of the commercial HA (HA), C-HA (600 °C), C-biphasic calcium phosphate (BCP) (700 °C), C-BCP-1 (800 °C), C-BCP-2 (900 °C), and C-β-tricalcium phosphate (βTCP) (1000 °C).

CaPs originate from cuttlefish bones and are produced by hydrothermal treatment and calcination. A precursor composed of HA (Ca_10_(PO_4_)_6_(OH)_2_, PDF No. 09-0432) and a small amount of monetite (CaPO_3_(OH), PDF No. 09-0080) was obtained after the hydrothermal procedure. Subsequently, different phases of C-CaPs were generated depending on the calcination temperature (600, 700, 800, 900, 1000, and 1100 °C). As shown in Figure 2a, the precursor calcined at 600 °C yields HA with the maximum degree of phase stability. Afterwards, the BCP (C-BCP) bioceramic begins to form when the precursor calcines at 700 °C. The diffraction peak of the β-TCP (PDF No. 09-0169) appears progressively and exhibits a narrow and sharp changing trend with an increase in the calcination temperature, suggesting that HA gradually decomposes into β-TCP as the temperature increases. According to a previous study, HA and β-TCP react mutually as follows [23]:

(2) $${Ca}_{10}{\left({\mathrm{PO}}_{4}\right)}_{6}{\left(\mathrm{OH}\right)}_{2}↔3{Ca}_{3}{\left({\mathrm{PO}}_{4}\right)}_{2}+{\mathrm{Ca}}^{2+}+{2\mathrm{OH}}^{-}$$

The relevant contents of β-TCP and HA in this study are presented in Table 2. The amount of β-TCP in the C-BCP increased from 15% to 70% as the second-stage sintering temperature increased from 700 to 900 °C. In theory, hydroxyapatite (HA) can partially decompose into βTCP when the calcination temperature is higher than 1100 °C [24]. In this study, the precursor was completely converted into C-βTCP bioceramics as the sintering temperature approached 1000 °C. One of the reasons is ascribed to the fact that the obtained precursor also contains calcium-deficient hydroxyapatite (CDHA). CDHA has the same crystal structure as stoichiometric HA, but its thermal stability is significantly poorer, and its decomposition temperature is far lower [24]. CDHA can decompose into β-TCP when calcined at or above 700 °C [25,26]. The other reason is that the substitution of Ca^2+^ with trace elements will result in crystal lattice distortion and thermal instability, which can lower the decomposition temperature during the calcination process [3]

Figure 2b shows the FTIR spectra of different formulations of calcium phosphate in the bioceramics derived from cuttlefish bones. The characteristic bands corresponding to the phosphate (${\mathrm{PO}}_{4}^{3-}$) are observed in the ranges of 560–610 and 950–1100 cm^−1^. In this study, the bands at 1100 and 964 cm^−1^ were assigned to the stretching vibrations, and the bands at 577 and 605 cm^−1^ were related to the deformation vibrations of the ${\mathrm{PO}}_{4}^{3-}$ ions. Furthermore, the bands at 3570 and 632 cm^−1^, which appeared for the C-HA, C-BCP, C-BCP-1, and C-BCP-2 groups, were assigned to OH^−^ vibrations and stretching bands of the HA structure. These absorption peaks coincide with the characteristic peaks in the commercial HA spectrum and agree well with the reported HA reference spectrum [27,28,29]. Increasing the calcination temperature to 1000 °C led to the dehydroxylation of HA into the β-TCP phase and the disappearance of the bands at 3570 and 632 cm^−1^.

### 3.2. Morphology

**Figure 3:**
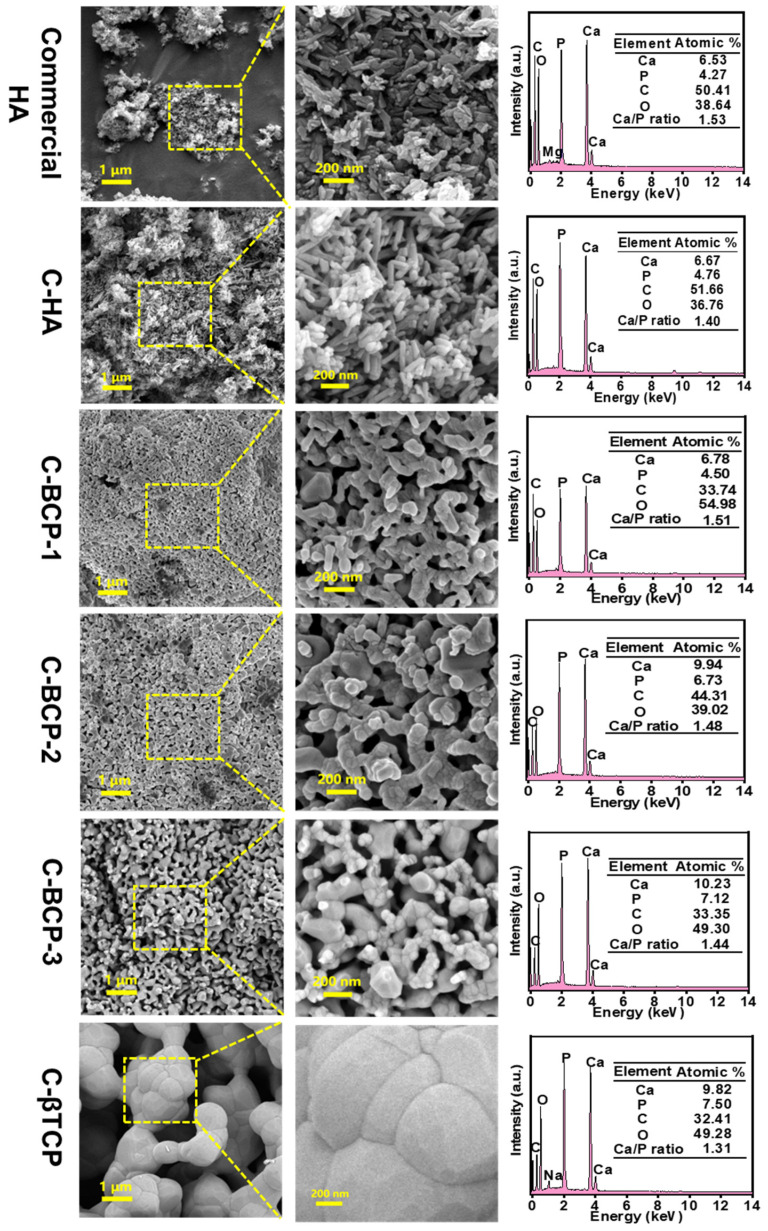
SEM images and EDS spectra of commercial HA, C-HA, C-BCP, C-BCP-1, C-BCP-2, and C-βTCP.

Figure 3 shows the variations in the morphologies and Ca/P ratios of the different samples. After calcination at 600 °C, the obtained C-HA had a stick-like shape, similar to the morphology of commercial HA. The particle-size distribution was in the range of 160–200 nm. EDS indicated that commercial HA had a Ca/P ratio of 1.53 (atomic ratio), which is lower than the ideal Ca/P ratio (1.67) for stoichiometric HA. This was primarily due to the incorporation of functional ions (such as Mg^2+^) into the commercial HA product. Furthermore, the Ca/P ratio of C-HA was lower than the stoichiometric ratio (1.67). This slight deviation may have been due to the formation of the trace elements and calcium-deficient HA [30].

The presence of trace ions in natural organic sources also contributes to the variance in the Ca/P molar ratio [16]. As the sintering temperature increased, the C-BCP grains became well -joined and exhibited a porous surface structure, suggesting that small granules agglomerated and developed into larger granules. The Ca/P ratio of C-BCP was ~1.50. Similar results were obtained in previous studies. The Ca/P ratios of BCP derived from Labeo rohita [31] and Salmo salar fish [24] bones were approximately 1.47 and 1.51, respectively. Furthermore, considerable changes in grain size and shape occurred in C-βTCP, which is attributed to microcrystals agglomerating into larger crystal grains after 1000 °C calcination.

### 3.3. Degradation and Ca^2+^ Dissolution Properties

**Figure 4:**
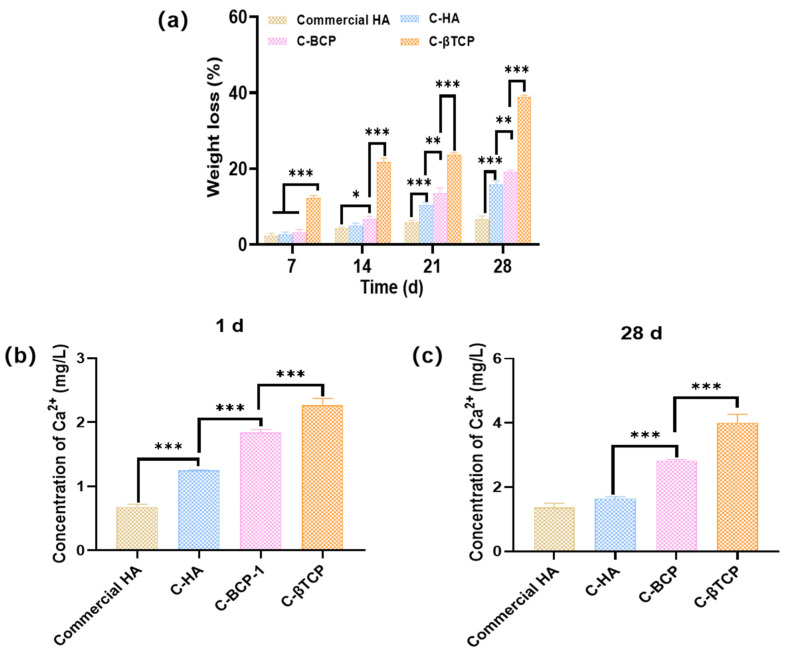
Degradation behavior of the commercial HA, C-HA, C-BCP, and C-βTCP after PBS immersion. (**a**) Weight loss of different samples as a function of the degradation time. (**b**) Ca^2+^ concentrations released from different samples after PBS immersion for 1 d. (**c**) Ca^2+^ concentrations released from different samples after PBS immersion for 28 d (* *p* < 0.05, ** *p* < 0.01, *** *p* < 0.001).

Degradability plays an important role in bone repair. The degradation behaviors of the samples were evaluated by measuring their weight loss in PBS solutions, and the results are shown in Figure 4a. The weight of all the samples decreased with an increase in the immersion time, and CaPs derived from cuttlefish bone exhibited a higher degradation rate than commercial HA. The biodegradability of CaPs is strongly related to their composition [19]. In this study, as β-TCP is more soluble than HA, the degree of dissolution for the C-CaPs decreased in the order of C-βTCP > C-BCP > C-HA. After immersion for 28 d, the weight losses of the commercial HA, C-HA, C-BCP, and C-βTCP were 6.78% ± 0.89%, 15.78% ± 1.11%, 19.11% ± 0.56%, and 38.89% ± 0.56%, respectively (Figure 4, Table S1). In addition, the ICP assay was employed to investigate the elemental compositions of the C-CaPs (Table S2). The findings indicated that the bioceramics had larger amounts of calcium and strontium but smaller amounts of potassium, sodium, and magnesium. Therefore, the concentrations of Ca^2+^ and Sr^2+^ that were released from different samples were measured using the ICP method. As shown in Figure 4 and Figure S2, the concentrations of Ca^2+^ and Sr^2+^ released by C-βTCP were higher than those of the other samples after 1 d of degradation, and after 28 d, the total concentration of Ca^2+^ reached 4.00 mg/L. The pH changes of commercial HA, C-HA, C-BCP, and C-βTCP from day 1 to day 28 are presented in Figure S1. There is no significant difference among the samples.

### 3.4. Apatite Formation Ability

**Figure 5:**
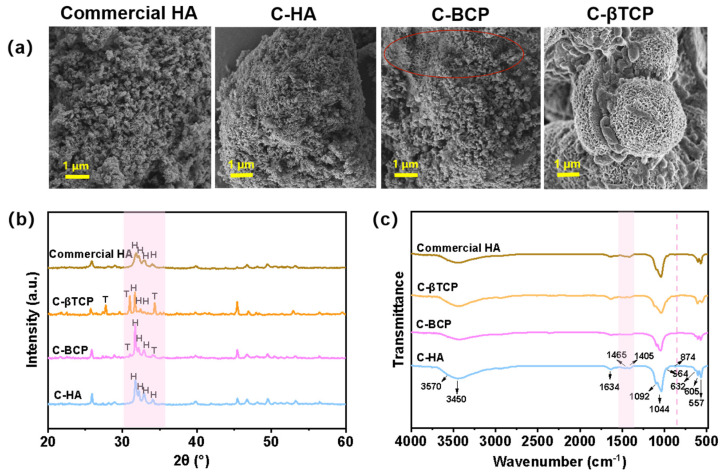
Bioactivity of the commercial HA, C-HA, C-BCP, and C-βTCP after SBF immersion for 14 d. (**a**) SEM images taken after SBF immersion for 14 d. (**b**) XRD and (**c**) FTIR spectra taken after SBF immersion for 14 d.

Mineralization was investigated by evaluating apatite formation on sample surfaces in SBF for 14 d, and the corresponding morphologies were examined via SEM (Figure 5a). A cauliflower-shaped morphology that comprised apatite nuclei consisting of short microstems was observed, along with clusters of these nuclei, which are characteristic of apatite nucleation and growth in SBF solutions [32]. The nucleation and growth of apatite on the sample occurred with the dissolution of its surface and precipitation of the apatite layer [33]. XRD was used to determine the phase changes of the deposits after SBF immersion for 14 d, and bioceramics without SBF induction served as controls (Figure 5b). The intensities of the characteristic peaks of β-TCP for C-βTCP were significantly reduced, while the characteristic peaks at 2θ = 31.76°, 32.18°, and 32.92°, which are ascribed to the diffraction planes (211), (112), and (300), respectively, of HA, appeared with high relative intensities. Furthermore, for the C-HA and C-BCP groups, the relative intensities of the HA peaks were increased, as well as the peak width. However, commercial HA exhibited no significant differences from the results obtained prior to SBF treatment. These findings indicate that the bone-like apatite formation ability of cuttlefish bone-derived bioceramics is stronger than that of commercial HA. This is attributed to the faster and stronger ion dissolution behavior of the cuttlefish bone-derived bioceramics, which can promote local saturation of apatite growth species (Ca^2+^/${\mathrm{PO}}_{4}^{3-}$) and function as apatite nucleation sites. FTIR spectroscopy was employed to further identify the functional groups present in the various samples (Figure 5c). The bands at 874 and 1448 cm^−1^ corresponding to typical ${\mathrm{CO}}_{3}^{2-}$ vibrations confirmed that carbonate hydroxyapatite had precipitated on all the bioceramic surfaces [34,35]. Together, the results indicated that cuttlefish bone-derived C-βTCP bioceramics significantly promoted bone-like apatite formation.

### 3.5. Cytotoxicity Assessment

**Figure 6:**
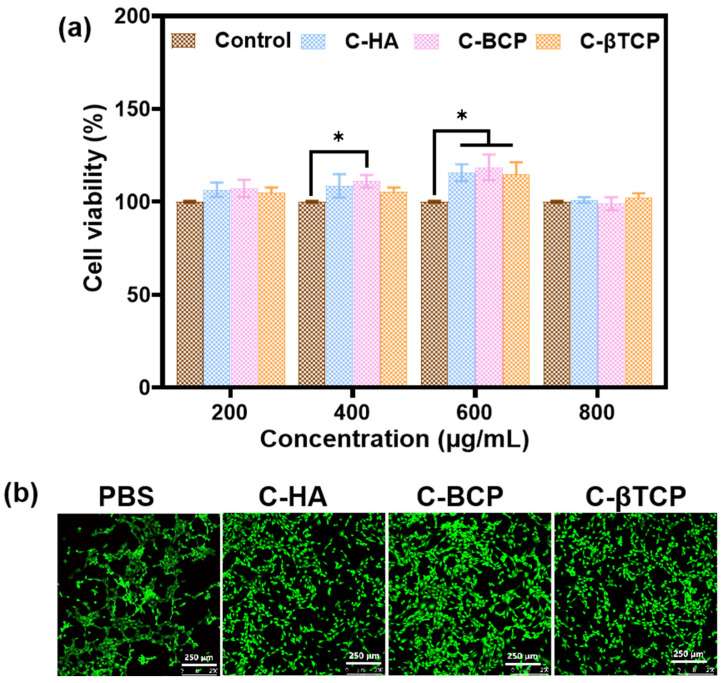
In vitro cytocompatibility of cuttlefish bone-derived CaPs. (**a**) Viability of cells with different concentrations of bioceramics. (**b**) Live/dead staining of cells on different samples. (* *p* < 0.05).

The CCK-8 assay, shown in Figure 6a, was employed to quantitatively assess the cell proliferation capacity of C-HA, C-BCP, and C-βTCP samples. According to ISO 10993-5 standard, a substance is nontoxic if the cell viability is >70% [36]. The results indicated that the cells were metabolically active when they were subjected to bioceramics at concentrations of 200, 400, 600, and 800 µg/mL. Furthermore, the live/dead staining results (Figure 6b) agree with those of the quantitative evaluation, indicating that the cuttlefish bone-derived bioceramics are biocompatible. The aforementioned findings indicate that C-CaPs have good biocompatibility and potential for a wide range of applications in bone tissue engineering.

### 3.6. Osteoblast Differentiation of rBMSCs In Vitro

**Figure 7:**
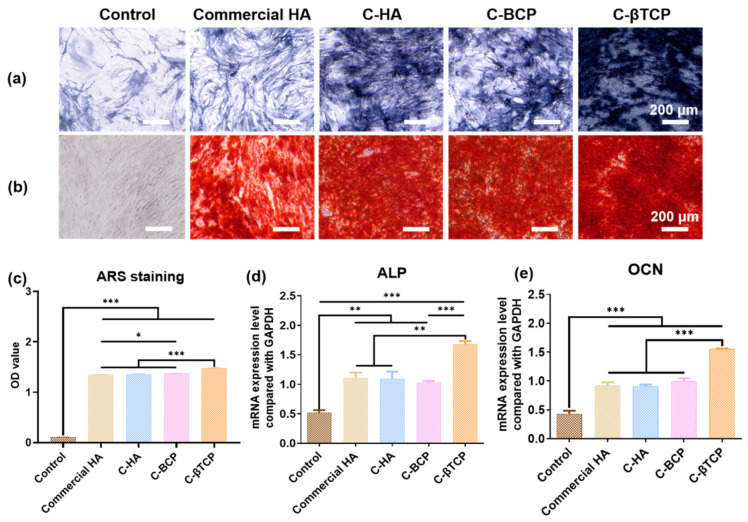
In vitro osteogenic induction evaluation of cuttlefish bone-derived CaPs. (**a**) ALP staining images of cells on the commercial HA, C-HA, C-BCP, and C-βTCP. (**b**) Alizarin Red S staining was performed on day 14, and (**c**) quantification was performed. The gene expression related to osteogenesis was evaluated via qRT-PCR. (**d**) ALP and (**e**) OCN levels were measured on day 14 for osteogenesis (* *p* < 0.05, ** *p* < 0.01, *** *p* < 0.001).

ALP is one of the essential markers of early osteogenesis [37]. ALP staining was performed to study the osteogenic potential of the C-CaPs. The ALP activity was significantly increased in the C-HA, C-BCP, and C-βTCP groups compared with commercial HA, indicating that the C-CaPs promoted rBMSC differentiation (Figure 7a). C-βTCP exhibited a higher degradation rate than the other bioceramics, corresponding to a higher ion dissolution capacity. Thus, for the C-βTCP group, the highest Ca^2+^ content contributed to the highest ALP activity. Calcium deposition is a marker of osteocyte maturation. Figure 7b,c show the results of Alizarin Red staining and the corresponding quantitative analyses. The red region represents the degree of cell differentiation. The color is deeper for the C-HA, C-BCP, and C-βTCP groups, indicating that cuttlefish bone-derived bioceramics have an advantage in calcium deposition. In particular, cells in the C-βTCP group exhibit a higher level of calcium deposition than those in the C-HA and C-BCP groups. This finding may also be related to the high Ca^2+^ content, which promotes rBMSC differentiation [38,39]. Osteogenic differentiation of rBMSCs was further evaluated by measuring the mRNA expression levels of ALP and OCN. The results indicated that the ALP gene markers were significantly upregulated in the rBMSCs cultured in the C-βTCP group and that there were no significant differences among the commercial HA, C-HA, and C-BCP groups on day 14 (Figure 7d). Moreover, OCN mineralization-specific genes were evidently upregulated in cells cultured in the C-βTCP groups compared with the other groups (Figure 7e). The C-βTCP group exhibited the highest expression levels of ALP (an early osteogenic marker) and OCN (a mature osteogenic marker), suggesting that it possessed the most capable compound for inducing and accelerating the osteo-differentiation of rBMSCs. According to our findings, as βTCP is more soluble than HA, the degree of dissolution for the C-CaPs decreases in the order of C-βTCP > C-BCP > C-HA. Therefore, the Ca^2+^ and Sr^2+^ concentrations released from C-βTCP were higher than those for the other samples. Additionally, the C-βTCP group had the highest expression level of osteogenic-related genes, which confirms the effective role of Ca^2+^ and Sr^2+^ in the osteogenesis process. Previous studies indicated that strontium-doped HA alters the crystal lattice, introducing defects that enhance bioactivity and promote bone repair [40]. Additionally, studies indicated that incorporating strontium into the HA structure improves its biological performance by stimulating osteoblast proliferation and differentiation while inhibiting osteoclast activity [41]. These findings suggest that C-βTCP is superior to commercial HA products for promoting for osteoinductive formation of new bone when implanted in vivo, which makes it promising for biomedical applications.

## 4. Conclusions

CaPs derived from natural cuttlefish bone were generated using a two-stage hydrothermal and calcination process. Different types of CaPs were obtained depending on the calcination temperature in the second stage. According to our findings, C-HA starts to transform into biphasic material at 700 °C and completely transforms into β-TCP at 1000 °C. Compared with commercial HA products, cuttlefish bone-derived bioceramics exhibit better degradation and ion dissolution properties, resulting in a higher apatite formation capacity and superior osteogenic differentiation ability. Among the extracted bioceramics, C-βTCP has the highest degradation rate, corresponding to the highest Ca^2+^ and Sr^2+^ dissolution capacity; thus, it significantly enhances bone-like apatite formation and rBMSCs osteogenic differentiation in vitro. Therefore, C-βTCP originating from cuttlefish bone is a promising raw bioceramic material for implant coatings and bone substitute scaffolds, with potential clinical applications.

In general, our research found that all bioceramics derived from cuttlefish bones, notably C-βTCP, have a higher degradation capacity, superior bone-like apatite formation ability, and osteogenic differentiation than commercially available hydroxyapatite. To further understand the role of C-βTCP in bone regeneration, a biodegradable composite scaffold containing C-βTCP and polycaprolactone (PCL) was produced using 3D printing technology. Future research will concentrate on assessing the osteogenic performance of this novel scaffold in comparison with some commercial bone scaffolds. Moreover, our future research should focus on in vivo investigations using different damage models of bone fracture to confirm their biocompatibility and therapeutic application potential.

## Figures and Tables

**Table 1:** Primers used in the RT-PCR.

Gene	Direction	Primer Sequence (5′ to 3′)
*m-GAPDH*	Forward	TGAAGCAGGCATCTGAGGG
	Reverse	TGAAGTCGCAGGAGACAACC
*m-OCN*	Forward	CAGTCCCCAGCCCAGAT
	Reverse	GTGATACCATAGATGCGTTTGT
*m-ALP*	Forward	TAAACCCTTCAGCCCTTCC
	Reverse	CCCTTGATACAGCACCTACATT

**Table 2:** Compositions of bioceramics prepared at various calcination temperatures.

Sample	Calcination Temperature (°C)	β-Tricalcium Phosphate (βTCP)%	Hydroxyapatite (HA)%
C-HA	600	0	100
C-BCP	700	15	85
C-BCP-1	800	50	50
C-BCP-2	900	70	30
C-βTCP	1000	100	0
C-βTCP-1	1100	100	0

## Data Availability

The raw data supporting the conclusions of this article will be made available by the authors upon request.

## Supplementary Materials

The following supporting information can be downloaded at: https://www.mdpi.com/article/10.3390/jfb15080212/s1, Figure S1: The pH change of the samples immersed in PBS solution; Figure S2: Sr^2+^ concentrations released from different samples after PBS immersion for 1 day. (*** *p* < 0.01); Table S1: The loss of mass of Calcium phosphate bioceramics; Table S2: Chemical composition determined by ICP-AES analysis.
