# Supplementary material for: Cuttlefish Bone-Derived Calcium Phosphate Bioceramics Have Enhanced Osteogenic Properties
Source: J Funct Biomater. 2024 Jul 29;15(8):212. doi: 10.3390/jfb15080212 (PMC11355212; doi:10.3390/jfb15080212)
Supplement: Supplementary file 1 [file jfb-15-00212-s001.zip › jfb-3070239-supplementary.PDF]

Supporting Information

Cuttlefish Bone-derived Calcium Phosphate Bioceramics Have Enhanced Osteogenic Properties

Boqi Pang[1,2,#], Jiaru Xian[1,2,#], Jiajun Chen[1], Liqi Ng[2], Mengting Li[1,*], Guangchun Zhao[1], Yixun E[1],Xiaorui Wang[1], Xiaxin Cao[1], Changze Zhang[1], Mingjing Zhang[2], Chaozong Liu[1,2,*]

[1] Hainan Provincial Fine Chemical Engineering Research Center, Hainan University, Haikou, 570228, P.R. China; 23220856020005@hainanu.edu.cn (B.P.); xianjiaru1234@163.com (J.X.); 20223003309@hainanu.edu.cn (J.C.); liqi.ng.17@ucl.ac.uk (L.N.); 23210817000033@hainanu.edu.cn (G.Z.); 20223003258@hainan.edu.cn (Y.E.); 20233003060@hainan.edu.cn (X.W.) ; 20203105022@hainanu.edu.cn (X.C.); hssyzcz@126.com (C.Z.); mingjing.zhang.22@ucl.ac.uk (M.Z.)

[2] Institute of Orthopaedic & Musculoskeletal Science, University College London, Royal National Orthopaedic Hospital, London, HA7 4LP, UK;

[#] These authors contributed equally to this work.

* Correspondence: limengting@hainanu.edu.cn (M.L.); chaozong.liu@ucl.ac.uk (C.L.)

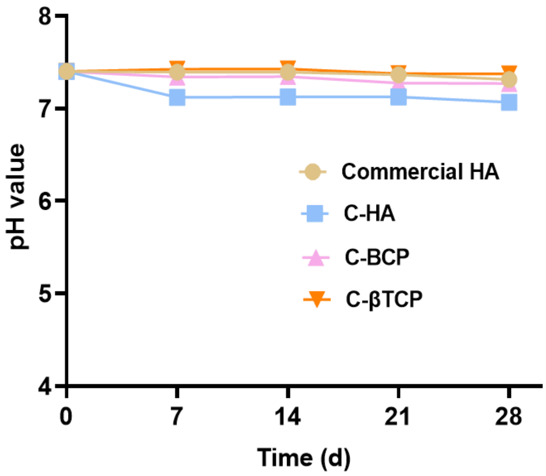

Figure S1. The pH change of the samples immersed in PBS solution.

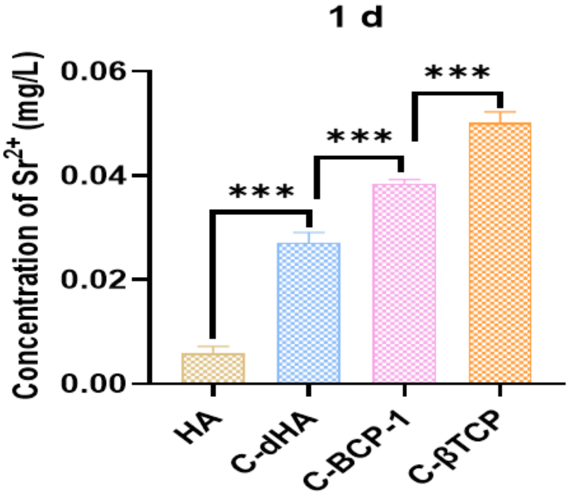

Figure S2. Sr^{2+} concentrations released from different samples after PBS immersion for 1 day.(***P < 0.01)

Table S1. The loss of mass of Calcium phosphate bioceramics.

Time（d）	HA（%）	C-HA（%）	C-BCP（%）	C-βTCP（%）
7	2.33±0.66	2.67±0.67	3.33±0.67	12.33±0.67
14	4.22±0.45	5.00±0.67	6.78±0.78	21.78±1.11
21	5.89±0.44	10.45±0.78	13.55±1.45	23.66±0.67
28	6.78±0.89	15.78±1.11	19.11±0.56	38.89±0.56

Table S2. Chemical composition determined by ICP-AES analysis.

Element	BCP(wt%)
Ca	38.4789
P	19.8896
Sr	0.2254
Na	0.1687
K	0.0924
S	0.0462
Mg	0.0124
Al	0.0102
Si	0.0099
Fe	0.0037
Zn	0.0033
Cu	0.0033
